# Predictive diagnostics and personalized medicine for the prevention of chronic degenerative diseases

**DOI:** 10.1186/1742-4933-7-S1-S1

**Published:** 2010-12-16

**Authors:** Federico Licastro, Calogero Caruso

**Affiliations:** 1 Department of Experimental Pathology, University of Bologna, Via San Giacomo 14, 40126, Bologna, Italy; 2 Immunosenescence Unit, Department of Pathobiology and Medical and Forensic Biotechnologies, University of Palermo, Corso Tukory 211, Palermo, Italy

## Abstract

Progressive increase of mean age and life expectancy in both industrialized and emerging societies parallels an increment of chronic degenerative diseases (CDD) such as cancer, cardiovascular, autoimmune or neurodegenerative diseases among the elderly. CDD are of complex diagnosis, difficult to treat and absorbing an increasing proportion in the health care budgets worldwide. However, recent development in modern medicine especially in genetics, proteomics, and informatics is leading to the discovery of biomarkers associated with different CDD that can be used as indicator of disease’s risk in healthy subjects. Therefore, predictive medicine is merging and medical doctors may for the first time anticipate the deleterious effect of CDD and use markers to identify persons with high risk of developing a given CDD before the clinical manifestation of the diseases. This innovative approach may offer substantial advantages, since the promise of personalized medicine is to preserve individual health in people with high risk by starting early treatment or prevention protocols. The pathway is now open, however the road to an effective personalized medicine is still long, several (diagnostic) predictive instruments for different CDD are under development, some ethical issues have to be solved. Operative proposals for the heath care systems are now needed to verify potential benefits of predictive medicine in the clinical practice. In fact, predictive diagnostics, personalized medicine and personalized therapy have the potential of changing classical approaches of modern medicine to CDD.

## Introduction

A dramatic increase in mean life span and life expectancy, coupled with a significant reduction in early mortality, has lead to a substantial increment in the number of the elderly population in contemporary societies. This demographic picture parallels the merging of a new epidemic characterized by chronic age related or degenerative diseases (CDD). Clinical diagnosis and therapy of these diseases imply multidisciplinary medical approaches and their cost is progressively increasing. Most CDD have complex aetiology and pathogenesis. In fact, human CDD are often the consequence of a complex interplay of genetic, epigenetic and environmental factors. Moreover current therapies are administered uniformly across a heterogeneous spectrum of disease aetiology, severity and genetic background. However, as large scale investigation platform become a mainstream, modern medicine has the opportunity to expand its medical instrumentation to include molecular phenotypes, genetic background and biomarkers to distinguish clinical subtypes of a single disease to better tailor both potential prevention strategies and/or early intervention protocols for CDD.

Therefore, the future treatment of CDD may contemplate primary, secondary or tertiary prevention. Primary prevention reduces diseases in a population by lowering exposure to casual agents or promoting host resistance and may lead to a decrease in age specific incidence rate or in a rise in the mean age at onset of a given CDD. Secondary prevention limits progression and recurrence of a disease by early case detection, diagnosis and treatment and reduces the disease prevalence. Tertiary prevention ameliorates the symptoms and tissue damages associated to a given CDD by containing individual disability and dependency and maintaining an acceptable quality of life.

Predictive diagnostics are merging from the acquisition of new phenotypic and genetic markers associated with CDD. This markers are generated by a panomic approach to biology of diseases such as genome wide association study, transcriptomics, proteomics and metabolomics. These disciplines attempt to capture the large output of an organism DNA, RNA, proteins and metabolites with the view that the whole is often greater than the sum of its parts and subtle critical information may be obtained by these methodological sophisticated approaches [1,2]. Recent advances in computational analysis has increased data availability for predictive medicine of CDD [3].

It is known that individuals are distinguished form one another by 0.1% difference in the nucleotide sequence of human genome. These difference are often in the form of a single base pair or single nucleotide polymorphism (SNP). Individual SNP usually causes only a modest change in the cognate protein concentration or function and is of limited information for disease’s risk evaluation. Therefore, the concomitant presence of several SNPs is more informative to determine susceptibility to disease development in polygenic human condition such as CDD. Finally, in complex genetic diseases the genotype confer susceptibility, but the clinical presentation of the disease depends upon interactions of several different genes with environmental factors.

Proteomic studies may then confirming and extending the findings of functional genomic investigations and provide novel markers for CDD.

Complementary to this global, non-hypothesis-driven methodologies, the candidate gene or marker approach evaluates individual marker in association with CDD based on a prior understanding of its role in the disease.

Genetic investigations on CDD are collecting useful information regarding genes associated with the disease risk. The goal will be to identify non symptomatic individual with high susceptibility for the disease who may benefit from prevention or early intervention protocols [4,5].

Current risk evaluation is derived from epidemiology investigation upon large populations and assesses the association of a trait or factor with the clinical outcome (disease manifestation, progression, etc.). Predictive medicine will modify this approach by introducing genomic and proteomic profile upon epidemiological investigation [6,7]. A marker is clinically useful if it predicts an event or a non-event in 70% of cases. To reach this goal a strong association between the marker and a given disease is required with an unadjusted relative risk or odds ratio = or > 10.0 [8]. A network of genetic, phenotypic, epidemiological and clinical factors may reach or go above that level of association and be very informative for the evaluation of individual risk for a given CDD.

One area of near future clinical application of these innovative methodologies is pharmacotherapy with the goal of optimizing the therapeutic response and minimizing toxicity and undesired effects [9,10]. For instance, Transtuzumab, a monoclonal antibody specific for HER2 receptor are only effective in women with HER2 positive breast cancer subtype [11]. Pharmacogenomic may also contribute to identify responders to a given drug and radically modify traditional pharmaceutical trials and disease’s treatment.

To discuss these topics a one day international meeting entitled “Predictive diagnostics and prevention of chronic degenerative diseases” was organized by Dr. Licastro at the Academy of Science of the Bologna University on December 4^th^ 2009, some of the contributes to the congress have been collected as full papers and presented in this special issue of Immunity and Aging.

The paper presented by Dr. Ravaglia and co-worker illustrate the relevance of longitudinal population study such as “the Conselice Study on Brain aging” to gather new markers linked to the risk of cognitive decline and dementia in old age and validate risk factors described in other epidemiological investigations.

Dr. Grossi presented a contribution illustrating a new statistical algorithm derived from artificial adaptive neural network systems for mining variables from biomedical database. This new statistical tool is able to describe a connectivity map of factors associated with a disease, the interactions among variables and their relevance with a given disease.

The article from Dr. Licastro and co-workers presented an application of the artificial adaptive system on a large number of variables collected from “the Conselice Study on Brain aging” longitudinal population investigation and showed a connectivity map of variables associated with the age associated cognitive decline and dementia.

Dr. Tomasi discussed the role of prion proteins on cellular kinases and their effects in signal transduction pathways of neurons in both physiological conditions and human neurodegneration.

Dr. Ianni and co-workers presented data regarding glycosilation profile of plasma purified alpha-1-antichymotrispin (ACT) in patients with Alzheimer’s diseases (AD) as a model of CDD. Data from previous investigations proposed ACT as peripheral marker associated with AD and Dr. Ianni’s presented paper suggests that a proportion of peripheral ACT may derive from AD brain, as indicated by the differential glycosilation profile of blood ACT from these patients.

Paper from Dr. Dogliotti and collaborators reported data from subjects with Down’s syndrome (DS), a complex human condition characterized by mental retardation, early presentation of Alzheimer’s disease, immune defects and increased susceptibility to infections. From this pilot investigation blood gelatinase is suggested as a candidate marker of inflammation associated with autoimmunity that is a frequent pathology in DS patients.
